# Vertical Force–Velocity Profiling in Soccer: A Systematic Review of Evidence, Assumptions, and Limitations

**DOI:** 10.3390/jfmk11010099

**Published:** 2026-02-27

**Authors:** Khairi Salim, El Mouahid Khalid, Chmura Paweł, Rfaki Abderrazak

**Affiliations:** 1 Department of Sports Training, Sports Science and Performance Optimization Research Team, Royal Institute for Managerial Training in Youth and Sport, Salé 11000, Morocco; salim.khairi213@gmail.com (K.S.); elmouahid_khalid@yahoo.fr (E.M.K.); rfakiabderrazak@gmail.com (R.A.); 2 Department of Individual and Team Sports, Wroclaw University of Health and Sport Sciences, 51-612 Wrocław, Poland

**Keywords:** force–velocity profile, neuromuscular performance, vertical jump, individualized training, team sports

## Abstract

**Background:** This systematic review critically examined how vertical force–velocity profiling has been used and interpreted in soccer research, with particular attention to methodological limitations and practical constraints. **Methods:** Following PRISMA guidelines, four databases were searched up to January 2025, and eleven studies met the inclusion criteria. **Results:** Several studies reported statistical associations between vertical F–V variables (particularly Pmax and V_0_) and jump- and sprint-related outcomes; however, these associations were heterogeneous, task-dependent, and sensitive to modeling assumptions. Age- and maturity-related studies demonstrate progressive increases in F_0_ and Pmax across developmental stages, explaining much of the inter-individual variability in youth populations. Positional and sex-based analyses reveal distinct neuromuscular profiles, with wide and attacking players displaying more velocity-oriented characteristics, and female players showing lower Pmax values. Indirect links with match-related demands, inferred from positional profiles and external load literature, suggest potential ecological relevance; however, direct evidence linking vertical F–V parameters to match-derived GPS metrics remains limited. Intervention studies show that individualized F–V-based training can modify selected vertical mechanical parameters, but improvements in sprint or match performance are not systematic. **Conclusions:** Vertical F–V profiling may provide descriptive information under tightly controlled conditions; however, evidence supporting its use for individualized or deficit-based training prescription in soccer remains limited and inconsistent. For this reason, vertical F–V profiling should not be interpreted as a mechanistic model of soccer performance, but rather as a context-dependent descriptive framework with restricted ecological validity.

## 1. Introduction

Soccer performance depends on a complex interaction between tactical demands, situational constraints, and the neuromuscular capacity to execute sequences of explosive actions such as acceleration, repeated sprints, jumps, changes in direction, and physical duels [1,2]. The vertical force–velocity (F–V) profile has been proposed as a methodological approach to describe jump performance through regression-derived parameters obtained from vertical jump tasks [3]. However, its mechanical validity, physiological interpretation, and applicability to soccer performance remain debated in the contemporary literature. This model typically provides estimates of theoretical maximal force (F_0_), theoretical maximal velocity (V_0_), maximal power (Pmax), and the F–V slope (Sfv), which should be interpreted as statistical descriptors of jump performance under constrained experimental conditions rather than as direct measures of neuromuscular or mechanical properties [4]. Recent research has increasingly questioned the mechanical validity, reproducibility, and physiological interpretation of vertical force–velocity profiling. Several studies have demonstrated that F–V parameters (F_0_, V_0_, and slope) are highly dependent on modeling assumptions, load selection, and measurement noise, and therefore should be considered regression-derived descriptors of jump performance rather than direct indicators of neuromuscular function [5,6,7]. In addition, inverse mathematical coupling between F_0_ and V_0_ and limited between-day reliability further challenge their use for individualized training prescription [8]. From a conceptual perspective, the relevance of vertical-jump-based F–V profiling for horizontally dominated sports such as soccer remains debated, and its transfer to sprint and match-related performance appears inconsistent and task-dependent [9,10].

Evidence suggests that force–velocity (F–V) parameters vary according to age group, sex, playing position, and competitive level. Studies conducted in youth athletes report significant increases in F_0_ and Pmax with biological maturation, whereas V_0_ exhibits more limited progression, indicating a differentiated development of force and velocity components [11,12]. In adult populations, elite players show variability in F–V profiles depending on playing position and competition level. For example, forwards and wingers generally display higher Pmax values, whereas goalkeepers tend to exhibit more velocity-oriented profiles reflecting the demands of rapid push-off actions [13,14]. Research further indicates that professional female players typically present lower Pmax values than their male counterparts, accompanied by steeper (more negative) F–V slopes. These differences have been attributed to morphological characteristics and disparities in exposure to resistance and power-oriented training programs [15]. However, these observed variations must be interpreted with caution. F–V parameters are highly dependent on testing protocols, load selection, and mathematical modeling strategies, which likely contribute to the heterogeneity of findings reported across studies [8].

The correlation between the vertical F–V profile and soccer-specific performance remains inconsistent. Numerous studies demonstrate a robust correlation between Pmax and jump height (r = 0.70–0.90), and between V_0_ and acceleration performance (r = 0.50–0.80), thereby validating the significance of these variables in predicting explosive actions [16,17]. On the other hand, F_0_ often has weaker or even inconsistent links to linear speed and horizontal performance measures. This substantiates the notion that vertical and horizontal mechanical properties are regulated by separate determinisms [18]. Importantly, soccer performance is dominated by horizontally oriented actions such as acceleration, deceleration, and change in direction. Therefore, the extent to which a vertical jump-based F–V model can meaningfully inform performance in a multidirectional and horizontally driven sport remains uncertain and requires critical examination.

Nonetheless, the literature demonstrates considerable methodological variability, including differences in jump tasks (squat jump vs. countermovement jump vs. loaded jumps), external loading strategies, measurement technologies (force platforms, linear encoders, optoelectronic systems), and mathematical modeling procedures used to derive F–V parameters [18,19]. Moreover, the majority of studies do not account for critical variables such as biological maturity, the menstrual cycle, training load, or inter-team variations in strength and conditioning methodologies, thereby complicating result comparisons and potentially undermining the external validity of conclusions [20,21,22,23].

In this context, it is essential to perform a systematic synthesis to assess the consistency of existing findings, evaluate the methodological rigor of available studies, and critically analyze the correlation between vertical F–V profile parameters and soccer performance [24]. Prior reviews have predominantly concentrated on speed, strength, or power in isolation, neglecting the comprehensive mechanical insights offered by the vertical F–V profile and failing to evaluate the risk of bias (RoB), which is crucial for assessing the overall quality and credibility of the evidence [25,26].

Several systematic reviews have examined explosive performance, sprint training, and power development in soccer and in sport more broadly. For example, Baena-Raya et al. synthesized evidence on vertical and horizontal force–velocity profiling in sports performance, highlighting its biomechanical relevance but without focusing specifically on soccer populations or on contextual determinants such as age, sex, and playing position [27]. More recently, Solberg et al. conducted a systematic review and meta-analysis on force–velocity-profile-based training to improve vertical jump performance across multiple sports, reporting potential benefits but without specifically targeting soccer players [10]. Importantly, none of these reviews critically examined the theoretical assumptions, methodological limitations, and task-specific relevance of vertical F–V profiling within soccer populations. In parallel, reviews on match demands in soccer based on GPS-derived metrics have emphasized large methodological variability in high-speed running and sprint thresholds, which further complicates ecological interpretation of laboratory-based neuromuscular profiles [28].

The novelty of the present systematic review lies in its exclusive focus on vertical force–velocity profiling in soccer players and its critical examination of this framework. Unlike previous reviews that primarily addressed sprint performance, jump training or general strength, and power development across multiple sports, this review synthesizes evidence on the determinants of the vertical F–V profile (age, biological maturation, sex and playing position) specifically within soccer populations.

Importantly, the present study explicitly considers methodological heterogeneity, risk of bias, and the conceptual limitations of the F–V framework, rather than assuming its mechanistic validity. By integrating these dimensions, this review provides a more cautious and critical appraisal of the practical relevance of vertical F–V profiling for performance assessment and individualized training prescription in soccer.

Consequently, a systematic review conforming to the PRISMA 2020 protocol is essential to elucidate the current state of knowledge, emphasize methodological constraints, and evaluate the potential usefulness and practical relevance of the vertical F–V profile as a tool for assessment and training individualization in football [29].

## 2. Materials and Methods

### 2.1. Experimental Approach

This systematic review was conducted and reported in accordance with the Preferred Reporting Items for Systematic Reviews and Meta-Analyses (PRISMA) 2020 guidelines [30]. The review protocol was also preemptively registered in the International Prospective Register of Systematic Reviews (PROSPERO), registration ID: CRD42025124706 [31]. A completed PRISMA 2020 checklist and the PRISMA flow diagram are provided as part of the manuscript.

### 2.2. Eligibility Criteria

The PICO framework initially posed the question: “In football players, how does the vertical force–velocity profile, assessed via vertical jump tests, correlate with neuromuscular performance variables (e.g., power, sprint, jump), and what are the methodological and practical implications for optimizing individualized training?” This method was utilized to evaluate the studies for eligibility. The PICO framework used to define the eligibility criteria is summarized in Table 1. The criteria for inclusion and exclusion were established beforehand [32].

### 2.3. Inclusion Criteria

We included only original peer-reviewed studies published in English between January 2015 and April 2025. Eligible studies involved male or female association soccer players across all competitive levels (youth, amateur, professional, or elite). Studies had to assess the vertical force–velocity (F–V) profile using vertical jump tests (squat jump, countermovement jump, or loaded jumps) and report at least one mechanical parameter (F_0_, V_0_, Pmax, or F–V slope). Included studies examined associations with neuromuscular performance outcomes such as jump height, sprint speed, or power output. Experimental, observational, and correlational designs using validated measurement instruments (e.g., force plates, linear encoders, motion capture systems or contact mats) were eligible, provided that sufficient methodological rigor and transparent statistical reporting were ensured. Only full-text articles with accessible data and compliance with ethical research standards were included in the qualitative synthesis.

### 2.4. Exclusion Criteria

Studies were excluded if they involved non-soccer populations (e.g., American football or other team sports), focused exclusively on horizontal force–velocity profiling, isokinetic testing or did not report quantitative vertical mechanical parameters (F_0_, V_0_, Pmax, or F–V slope). Reviews, meta-analyses, conference abstracts, editorials, and non-peer-reviewed articles were excluded. Studies lacking full-text availability, sufficient methodological detail, or clearly defined data acquisition protocols were also excluded to ensure scientific comparability and validity of the synthesis.

### 2.5. Information Source

SCOPUS, Web of Science and Science Direct were selected for the literature review.

### 2.6. Search Strategy

A comprehensive literature search was conducted in SCOPUS, Web of Science, PubMed and Science Direct. The search was performed between April and August 2025, with the last search conducted on 15 April 2025. Studies published between January 2015 and April 2025 were considered eligible.

The search strategy for Scopus was as follows:

(“football” OR “soccer”) AND (“Force-velocity profile” OR “Vertical force-velocity profiling” OR “Vertical force-velocity profile” OR “F-V profile” OR “FVP”).

Equivalent search strategies were adapted for Web of Science, Science Direct and PubMed. Filters were applied for English language and peer-reviewed journal articles.

The reference lists of included studies were manually screened to identify additional potentially relevant articles. No additional eligible studies were identified beyond those retrieved through the database search. Full search strategies for each database are provided in the Supplementary File S1 in accordance with PRISMA-S recommendations.

### 2.7. Study Selection Procedure

Study selection was performed independently by two reviewers (S.K. and K.E.M.). Titles and abstracts were first screened according to the predefined eligibility criteria. Full-text articles of potentially relevant studies were then retrieved and assessed for eligibility. Disagreements between reviewers were resolved through discussion and consensus. When necessary, a third reviewer (A.R.) was consulted.

After removal of duplicates, 176 records were screened by title and abstract. Of these, 131 records were excluded because they did not meet the inclusion criteria (non-soccer populations, horizontal force–velocity profiling only, review articles, or unrelated outcomes). Forty-five full-text articles were assessed for eligibility. Among these, 35 articles were excluded for the following reasons: non-soccer populations (*n* = 7), absence of vertical force–velocity mechanical parameters (*n* = 9), outcomes not related to neuromuscular performance (*n* = 6), inaccessible full texts (*n* = 5), and insufficient methodological detail (*n* = 8). Consequently, 10 studies were included in the qualitative synthesis. The study selection process is illustrated in the PRISMA 2020 flow diagram.

### 2.8. Date Extraction

The process of extracting and analyzing data was done using a strict method, which made sure that the results from the studies included were always and fully understood. A standardized grid was used to systematically gather relevant information, such as the name of the first author, the year of publication, the study design and sample size (N), the study population (age and sex), the key parameters of the force–velocity profile (F_0_, V_0_, Pmax, and FV slope) and all reported links to performance outcomes.

### 2.9. Risk-of-Bias Assessment

Given that the majority of the included studies were observational in design (cross-sectional or correlational) rather than interventional, the risk of bias was assessed using the Joanna Briggs Institute (JBI) Critical Appraisal Checklist for Analytical Cross-Sectional Studies. This tool is specifically designed to evaluate methodological quality in observational research and covers key domains including participant selection, measurement of exposure and outcomes, identification and control of confounding factors, and appropriateness of statistical analyses.

Two reviewers (S.K. and K.E.M.) independently assessed the methodological quality of each included study. Any disagreements were resolved through discussion and consensus, and when necessary, a third reviewer (A.R.) was consulted. Each study was classified according to the number of criteria fulfilled, allowing a qualitative interpretation of overall methodological rigor.

The results of the risk-of-bias assessment were considered in the interpretation of findings, particularly when discussing the strength of evidence and the heterogeneity of reported outcomes. No causal inferences were drawn from studies presenting important methodological limitations.

## 3. Results

**Figure 1:**
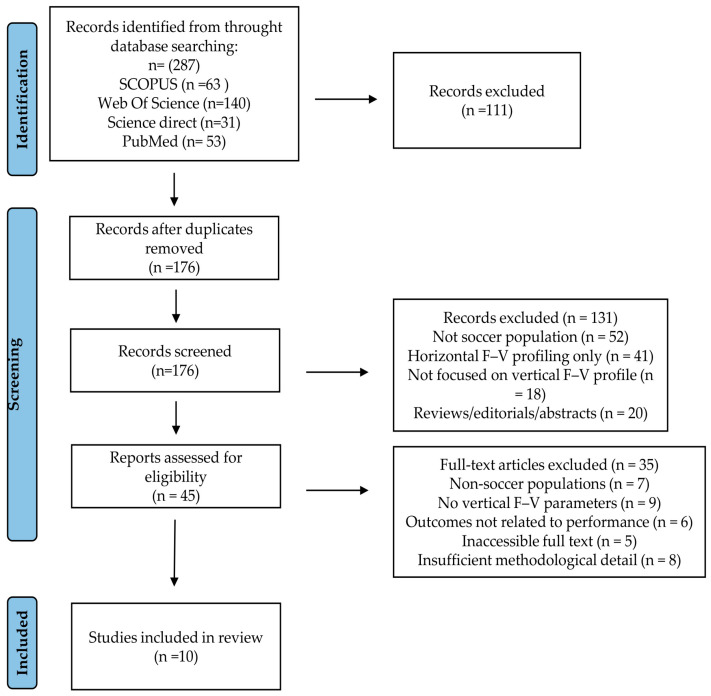
PRISMA 2020 flow diagram of the study selection process.

A comprehensive search of four electronic databases (Scopus, Web of Science, Science Direct, and PubMed) identified a total of 287 records (Scopus: *n* = 63; Web of Science: *n* = 140; ScienceDirect: *n* = 31; PubMed: *n* = 53). After removal of duplicates, 176 unique records remained for title and abstract screening. Of these, 131 records were excluded because they did not meet the inclusion criteria (non-soccer populations, horizontal force–velocity profiling only, review articles or unrelated outcomes). Forty-five full-text articles were assessed for eligibility. Among these, 35 studies were excluded for the following reasons: non-soccer populations (*n* = 7), absence of vertical force–velocity mechanical parameters (*n* = 9), outcomes not related to neuromuscular performance (*n* = 6), inaccessible full texts (*n* = 5) and insufficient methodological detail (*n* = 8). Consequently, 10 studies met all inclusion criteria and were included in the qualitative synthesis. The complete study selection process is presented in the PRISMA 2020 flow diagram Figure 1.

The ten included studies were categorized according to their primary research objectives and methodological design. Most studies were observational in nature and aimed to characterize the vertical force–velocity (F–V) profile in specific subpopulations of soccer players. These investigations examined the influence of factors such as biological maturity, competitive level, and playing position on vertical F–V parameters (F_0_, V_0_, Pmax, and F–V slope) [11,33,34,35,36,37,38]. In addition, several correlational studies explored the associations between vertical F–V profile variables and performance outcomes, including jump height and sprint performance [17,38,39].

Unlike previous reviews that included heterogeneous populations or intervention-based protocols, the present synthesis focused exclusively on soccer players and observational designs. This approach allowed for a clearer interpretation of the determinants and practical relevance of vertical F–V profiling within soccer-specific contexts, while avoiding confounding effects related to training interventions or non-soccer populations.

### Result Tables

The selected studies were systematically analyzed to summarize the main methodological characteristics, the mechanical parameters of the vertical force–velocity profile and the relationships associated with physical performance in soccer players.

The methodological characteristics of the included studies (Table 2) demonstrate substantial heterogeneity in participant populations, testing protocols (SJ, CMJ, multi-load), measurement devices, and modeling approaches. Given that force–velocity parameters are highly sensitive to task execution, load selection, and regression assumptions, this heterogeneity limits construct equivalence and reduces the comparability of results across studies. Consequently, between-study differences may partly reflect methodological variability rather than true neuromuscular distinctions, which constrains the interpretability of the synthesized findings.

The mechanical outcomes reported in Table 3 show apparent age- and protocol-dependent variations in vertical F–V parameters. However, these trends should be interpreted cautiously, as they may reflect scaling effects, regression coupling, and substantial heterogeneity in testing procedures rather than true neuromuscular differences. Intervention studies indicate task-specific modifications of F_0_ or V_0_, yet these changes are inconsistent and rarely translate into sprint or football-specific performance outcomes. Overall, the results are constrained by moderate-to-serious risk of bias and limited construct equivalence across studies.

The correlations and key findings summarized in Table 4 indicate that associations between vertical force–velocity variables and performance outcomes are highly context-dependent and heterogeneous across studies. Maximal power (Pmax) is the parameter most frequently reported as being associated with jump height, whereas relationships with acceleration or peak sprint velocity are inconsistent and vary according to population characteristics and testing protocols. Importantly, these associations should not be interpreted as evidence of a direct mechanistic link, as F_0_, V_0_, and F–V slope are mathematically interrelated and sensitive to modeling procedures, load selection, and normalization strategies. Several reported correlations appear restricted to specific subgroups (e.g., maturing athletes) and are not consistently observed across age categories or competitive levels. The absence of detectable menstrual cycle effects and the limited transferability between vertical and horizontal F–V profiles further support the task-specific nature of these parameters and question their generalizability to football-specific performance. Overall, the interpretation of these correlations is constrained by substantial methodological heterogeneity and the moderate-to-serious risk of bias identified in most included studies.

Table 5 summarizes descriptive averages (mean ± SD and ranges) of normalized vertical force–velocity profile parameters across different competitive levels in soccer players. These values were calculated as unweighted means from the contributing studies, and the number of studies included in each category is reported. No formal meta-analytic pooling was performed.

Table 6 shows a summary of the methodological quality of the studies that were included, as measured by the Joanna Briggs Institute (JBI) Critical Appraisal Checklist. In general, two studies were found to have a low risk of bias [11,37], while the other eight studies showed a moderate risk of bias. The principal sources of methodological limitations pertained to the identification and management of confounding variables, including biological maturity, training load, and strength-training history (Q5 and Q6). Conversely, the majority of studies demonstrated substantial methodological rigor in participant characterization (Q2), exposure and outcome assessment employing validated biomechanical protocols (Q3, Q4, Q7), and suitable statistical evaluations (Q8). These results suggest that, while vertical force–velocity profiling was typically evaluated through standardized and dependable methods, caution is necessary when interpreting correlations with performance outcomes due to the observational nature of the study and the restricted control over confounding variables.

## 4. Discussion

The findings of this systematic review indicate that the vertical force–velocity (F–V) profile may serve as a context-dependent descriptive framework of jump-related neuromuscular characteristics in soccer players rather than as a comprehensive or mechanistic model of soccer performance; however, the strength of evidence remains limited due to substantial methodological heterogeneity across studies [10,16,40,41], and conclusions must be interpreted with caution in light of the moderate-to-serious risk of bias identified in most included studies (Table 6) and the large variability in testing protocols (SJ vs. CMJ, load selection, measurement devices, and modeling approaches). Results consistently indicate that F_0_ and Pmax rise with biological maturation across various age groups, whereas V_0_ exhibits more variable patterns. Research investigating age- and maturity-related disparities [11,33,42] consistently demonstrates that post-PHV players display superior maximal force and power outputs compared to pre- and mid-PHV players. These maturational effects predominantly elucidate the inter-individual variability observed in youth populations and underscore that inadequate control for growth-related factors may exaggerate or obscure associations between F–V variables and performance. This corroborates prior longitudinal findings indicating that increases in maximum strength occur more rapidly than enhancements in speed-related attributes during adolescence [43].

When examining positional demands, the analyzed studies consistently demonstrate significant inter-role variations in F–V mechanical expression. Studies examining playing positions [17,44,45] reveal that wide and attacking players typically exhibit velocity-oriented profiles and elevated Pmax, corresponding to their frequent participation in high-speed and sprinting activities, whereas central defenders and defensive midfielders demonstrate force-oriented profiles. Goalkeepers constitute a unique subgroup [13]. Elite goalkeepers exhibit steeper force–velocity (F–V) slopes and elevated theoretical maximal velocity, indicating their necessity for swift, short-range explosive actions such as dives and reactive jumps. These results demonstrate that understanding F–V profiling necessitates taking into account the position and strategy of the game. Nevertheless, positional comparisons were derived from heterogeneous protocols (different jump tests, external loads, and devices), which limits direct quantitative comparison between studies and may partially explain inconsistencies in reported F–V patterns.

Sex-related differences in vertical F–V parameters were observed, although these patterns were not entirely consistent across studies. Research included in this review indicates that elite female athletes generally demonstrate lower Pmax and steeper F–V slopes in comparison to their male counterparts [15,17]. However, significant intra-group variability exists, with certain female athletes nearing the performance levels of sub-elite males. Study [38] showed that the phase of the menstrual cycle and the use of oral contraceptives do not have a significant effect on vertical F–V parameters. This suggests that training exposure and strength-power development more strongly influence biological sex-related variability than hormonal changes. These findings indicate that menstrual cycle phase does not appear to substantially affect vertical F–V parameters, which supports the feasibility of repeated neuromuscular monitoring in female soccer players. However, these conclusions are based on a limited number of studies with moderate risk of bias, and further standardized investigations are required before firm sex-specific recommendations can be made.

Associations between vertical F–V variables and selected performance outcomes were reported in several studies, although their magnitude and consistency varied. Importantly, these associations were derived from studies using different jump modalities (SJ vs. CMJ), load increments and modeling procedures, which likely contributed to the variability in correlation coefficients and precludes strong causal interpretation. Pmax and V_0_ exhibited the most robust correlations with sprint and jump performance [15,17,46], whereas F_0_ demonstrated weaker and more context-sensitive associations, especially during high-velocity tasks. Numerous studies have indicated that the functional role of F_0_ is contingent upon the mechanical constraints of the task; elevated correlations are observed during loaded jumps, early acceleration, or among players with force-oriented profiles [35,47]. In contrast, [39] presented robust evidence indicating that F_0_ and V_0_ are significantly task-specific and do not transfer across modalities (sprint, jump, hip thrust), thereby emphasizing that vertical F–V parameters should not be presumed to predict horizontally oriented performance. 

These mechanistic findings correspond with vector specificity theory [3]; explosive actions in football are regulated by direction-specific mechanical capabilities and highlight that vertical profiling captures only one component of multidirectional neuromuscular performance in soccer. Importantly, the strong task- and context-dependence of vertical F–V parameters challenges their interpretation as general indicators of football-specific neuromuscular function. F_0_, V_0_, and F–V slope are regression-derived descriptors that are mathematically interrelated and highly sensitive to modeling assumptions, load selection, and measurement noise. Consequently, classifications of force or velocity “deficits” may partly reflect mathematical artifacts rather than true neuromuscular impairments. These conceptual limitations undermine the validity of using vertical F–V profiling as a universal profiling tool for multidirectional and horizontally dominated sports such as soccer [7,9].

Although no included study directly examined match-derived GPS metrics, previous research on positional demands provides contextual support for interpreting vertical F–V characteristics in relation to external load profiles. Wide and attacking players consistently execute a higher number of high-speed runs, accelerations, decelerations, and high-metabolic-load actions (>25–30 km·h^−1^; >2–3 m·s^−2^), and players with higher V_0_ or Pmax may theoretically be better suited to repeated high-speed actions [28,37,48]. No included study directly linked F_0_ deficits to HMLD; however, evidence indicates that neuromuscular fatigue resulting from repeated accelerations and decelerations affects match load metrics [49,50]. Future studies integrating vertical F–V profiling with GPS-derived external load metrics are required to improve ecological validity and individualized training prescription.

Evidence for interventions is still limited and not always clear [19,51]. Some intervention studies reported improvements in vertical jump performance following individualized F–V-based training; however, these mechanical adaptations were not consistently accompanied by improvements in sprint performance. Recent experimental studies yield more nuanced interpretations. The study [52] demonstrated that six weeks of personalized training yielded significant improvements in V_0_ for velocity-deficit athletes (+21.9%) and moderate enhancements in CMJ height for force-deficit athletes (+4.2%). Importantly, these vertical mechanical improvements did not translate into sprint performance enhancement and were accompanied by a reduction in horizontal V_0_, highlighting a limited transfer to horizontally oriented tasks. However, these advancements did not result in sprint performance enhancement and were accompanied by a decrease in horizontal V_0_. Ref. [35] offered mechanistic guidance by demonstrating that light ballistic loading primarily impacts V_0_ (−9.7%), while heavy loading chiefly decreases F_0_ (−8.4%). This finding further indicates that vertical F–V parameters are highly task-specific and sensitive to acute neuromuscular fatigue, and that correcting isolated vertical force or velocity deficits may not be sufficient to improve multidimensional soccer performance. This indicates that vertical F–V parameters are highly sensitive to acute neuromuscular fatigue, suggesting that rectifying isolated deficits may not enhance multidimensional performance. Ref. [10] indicated that individualized programs only partially rectified force or velocity deficits and failed to surpass non-optimized training, whereas [5] discovered no significant advantage of individualized prescriptions in team-sport athletes. Together, these results emphasize that improvements in vertical F_0_, V_0_, or Pmax do not consistently translate into sprint or match-related performance gains. Overall, while individualized F–V-based interventions may induce specific neuromuscular adaptations in vertical tasks, their practical superiority over conventional training approaches and their transfer to sprint performance remain uncertain and should be interpreted with caution. This uncertainty is compounded by the moderate-to-serious risk of bias and the heterogeneity of intervention protocols, which limits the generalizability of current findings.

Methodological limitations across studies considerably undermine the robustness of the evidence. As highlighted by the Risk-of-Bias assessment (Table 6), most studies were classified as having moderate to serious risk of bias, mainly due to uncontrolled confounders and non-standardized testing procedures. Numerous studies are compromised by uncontrolled confounders such as maturity status, training exposure, positional demands, and accumulated fatigue, alongside small sample sizes, inconsistent load prescriptions, heterogeneous modeling procedures, and insufficient familiarization. Moreover, numerous studies utilized convenience sampling or retrospective peak-performance data, thereby exacerbating selection and reporting bias. Ref. [47] conducted a feasibility analysis indicating that vertical F–V profiling can be implemented under controlled conditions, although fatigue, congestion and equipment variability may introduce measurement inconsistencies that limit longitudinal monitoring, and testing conditions such as fatigue, congestion, and equipment variability may introduce inconsistencies that hinder longitudinal monitoring. Overall, the consistency in the direction of observed associations suggests that vertical F–V parameters may offer meaningful insights when methodological rigor is ensured.

### Limitations

Several limitations should be acknowledged. Considerable methodological heterogeneity was observed across studies with respect to jump test selection (SJ vs. CMJ), load magnitudes, measurement devices and modeling approaches, which limits comparability and precludes quantitative synthesis. In addition, most studies were characterized by small sample sizes and restricted populations, thereby reducing external validity. The overall risk of bias was moderate to serious, mainly due to observational designs and insufficient control of confounding variables such as biological maturity, training exposure, and positional demands. Furthermore, variability in participant characteristics and testing protocols introduces inconsistencies in inclusion and exclusion criteria across studies. Finally, reliance on vertical force–velocity profiling alone represents a conceptual limitation, as football performance is largely dependent on horizontally oriented actions, and improvements in vertical F–V parameters do not consistently translate into sprint performance.

## 5. Conclusions

In summary, this systematic review shows that vertical force–velocity (F–V) profiling is a widely used descriptive approach derived from vertical jump tasks, but its interpretation as a marker of neuromuscular function in soccer players remains limited by substantial methodological and conceptual constraints. The available evidence suggests that F_0_ and Pmax vary with biological maturation and competitive level, whereas associations between V_0_ and acceleration performance are inconsistent and highly task-dependent. These findings must be interpreted with caution given the moderate-to-serious risk of bias across studies, the large heterogeneity in testing protocols, and the strong dependence of F–V parameters on modeling assumptions and measurement conditions.

Consequently, vertical F–V profiling should not be considered a mechanistic or comprehensive assessment of football performance, but rather a context-specific and protocol-dependent descriptive framework. Its potential utility lies in complementing other performance assessments under carefully standardized and controlled conditions, rather than serving as a basis for deficit-based training prescriptions.

Future research should prioritize standardized testing procedures, direct evaluation of reliability and construct validity, and integration with horizontally oriented performance measures that better reflect the multidirectional demands of soccer. Large-scale, longitudinal, and well-controlled studies are required to determine whether vertical F–V profiling provides added value beyond traditional strength and performance testing and whether any observed adaptations translate meaningfully to sprint and match-related outcomes.

## Figures and Tables

**Table 1:** PICO framework used to define the eligibility criteria of the review.

P	I	C	O
Male and female soccer players	Assessment or training using the vertical force–velocity profile	Across levels, loads, or training interventions	Effects on power, sprint, and jump performance

**Table 2:** Methodological characteristics of the included studies.

Author (Year)	Study Design	Population (Level, Age, Sex, (*n*))	Anthropometrics	FV Profiling Method	Task Type (SJ, CMJ, Multi-Load)
Zghal et al., 2025 [32]	Comparative observational (maturity groups)	Male youth soccer players; *n* = 116 (PHV−2 to PHV+3 groups)	Body mass: 39.6–74.8 kg; Height: 149–179.8 cm	Samozino method (multi-load)	Multi-load SJ (Hmax, H30, H60)
Bouvier et al., 2025 [37]	Comparative observational (menstrual cycle vs. oral contraceptives)	Elite female players; (*n* = 34)	60.3 ± 5.2 kg; 165.5 ± 4.2 cm	Force plate + Samozino	Multi-phase SJ (follicular, ovulatory, luteal)
Li et al., 2024 [34]	Experimental (fatigue protocols: HLT vs. LLB)	Male players; (*n* = 12)	85.9 ± 13.3 kg; 179.1 ± 4.2 cm	Samozino (multi-load)	Multi-load SJ (0–90% BM), pre/post-fatigue
Galantine et al., 2024 [33]	Comparative observational (maturity)	Males: Mid-PHV (*n* = 31), Post-PHV (*n* = 30), Adults (*n* = 23)	56.9–72.4 kg; 1.68–1.78 m	Samozino method	Multi-load SJ
Ben Hassen et al., 2024 [36]	Cross-sectional (positional comparison)	Male players; (*n* = 90) (GK, CD, WD, CM, WM, F)	66.2–73.8 kg; 173.7–180.4 cm	Samozino (multi-load)	Unloaded SJ
Junge et al., 2021 [38]	Cross-sectional	Male players; (*n* = 26)	76.4 ± 5.5 kg; 181.8 ± 6.4 cm	Samozino (multi-load)	SJ (unloaded, +30 kg, +60 kg)
Manson et al., 2021 [15]	Correlational observational	Elite international female players; (*n* = 39)	64.7 ± 6.3 kg; 168.8 ± 6.0 cm	Samozino	Unloaded SJ and CMJ
Fernández-Galván et al., 2021 [11]	Cross-sectional	Male youth players U10–U18; (*n* = 89)	Body mass: 29.7–71.8 kg; Height: 129–177 cm	Samozino multi-load	CMJ (single-task)
Marcote-Pequeño et al., 2018 [17]	Cross-sectional	Adult female soccer players; *n* = 19; 23.4 ± 3.8 yrs	59.7 ± 4.7 kg; 166.4 ± 5.6 cm	Force plate + Samozino	Unloaded SJ
Hervéou et al., 2018 [35]	Cross-sectional	Male goalkeepers; (*n* = 11)	78.2 ± 8.5 kg; 182.5 ± 6.4 cm	Force plate + Samozino	Unloaded SJ and CMJ

*SJ: squat jump, CMJ: countermovement jump, FV: force–velocity, PHV: Peak Height Velocity, BM: body mass, HLT: High-Load Training fatigue protocol, LLB: Low-Load, High-Velocity fatigue protocol, FD: Force-Dominant training group, VD: Velocity-Dominant training group, WB: Well-Balanced training group, GK: goalkeeper, CD: Center-Back, WD: Wide Defender, CM: Center Midfielder, WM: Wide Midfielder, F: forward.*

**Table 3:** Mechanical outcomes of the vertical force–velocity profile (F_0_, V_0_, Pmax, Sfv).

Author(s)	F_0_ (Vertical) (N·kg^−1^)	V_0_ (Vertical) (m·s^−1^)	Pmax (Vertical) (W·kg^−1^)	F–V Slope (N·s·m^−1^·kg^−1^)
Zghal et al., 2025 [32]	PHV−1: 26.4 ± 4.7, PHV: 28.3 ± 4.0, PHV+1: 28.8 ± 4.7, PHV+2: 29.1 ± 3.3, PHV+3: 29.0 ± 2.9	PHV−1: 3.1 ± 0.7, PHV: 3.4 ± 1.0, PHV+1: 3.1 ± 0.9, PHV+2: 3.2 ± 0.8, PHV+3: 3.7 ± 0.8	PHV−1: 19.7 ± 2.6, PHV: 23.2 ± 5.7, PHV+1: 21.9 ± 4.3, PHV+2: 23.1 ± 4.6, PHV+3: 26.3 ± 3.3	PHV−1: −9.3 ± 3.6, PHV: −9.4 ± 3.6, PHV+1: −10.1 ± 4.0, PHV+2: −9.7 ± 3.1, PHV+3: −8.2 ± 2.0
Bouvier et al., 2025 [37]	Eumenorrheic: 32.2 ± 3.4 OC users: 33.1 ± 3.8	Eumenorrheic: 2.73 ± 0.24 OC users: 2.78 ± 0.27	Eumenorrheic: 21.9 ± 2.9 OC users: 22.7 ± 3.1	Eumenorrheic: −11.8 ± 1.9 OC users: −12.1 ± 2.2
Li et al., 2024 [34]	Pre: 48.2 ± 3.4 Post HLT: 44.7 ± 3.6 Post LLB: 47.8 ± 3.0	Pre: 2.46 ± 0.11 Post HLT: 2.33 ± 0.19 Post LLB: 2.23 ± 0.18	Pre: 29.7 ± 3.5 Post HLT: 26.0 ± 3.1 Post LLB: 26.6 ± 2.9	Pre: 19.7 ± 1.5 Post HLT: 19.4 ± 2.1 Post LLB: 21.6 ± 2.1
Galantine et al., 2024 [33]	Mid-PHV: 23.5 ± 3.5 Post-PHV: 24.8 ± 3.0 Adults: 25.3 ± 2.9	Mid-PHV: 3.1 ± 1.0 Post-PHV: 3.3 ± 0.8 Adults: 3.6 ± 0.8	Mid-PHV: 17.9 ± 4.6 Post-PHV: 20.4 ± 4.4 Adults: 22.5 ± 4.8	Mid-PHV: −8.4 ± 3.3 Post-PHV: −7.9 ± 2.3 Adults: −7.4 ± 2.0
Ben Hassen et al., 2024 [36]	GK: 30.7 ± 3.8 CD: 28.8 ± 3.8 WD: 30.8 ± 3.3 CM: 30.0 ± 4.2 WM: 31.1 ± 3.7 F: 31.0 ± 1.4	GK: 3.1 ± 0.7 CD: 3.8 ± 1.2 WD: 3.4 ± 0.5 CM: 3.4 ± 0.9 WM: 3.7 ± 0.9 F: 3.5 ± 0.6	GK: 23.0 ± 2.8 CD: 26.8 ± 5.6 WD: 26.3 ± 4.9 CM: 25.1 ± 3.8 WM: 28.5 ± 4.8 F: 27.1 ± 4.3	NR
Junge et al., 2021 [38]	37.8 ± 4.8	3.28 ± 0.60	30.6 ± 4.7	Not reported numerically for vertical SJ
Manson et al., 2021 [15]	35.8 ± 5.2	2.35 ± 0.45	20.6 ± 2.3	−16.0 ± 4.9
Fernández-Galván et al., 2021 [11]	22.4 ± 9.8	3.62 ± 1.08	20.3 ± 11.1	−45.0 ± 25.6
Marcote-Pequeño et al., 2018 [17]	33.52 ± 3.61	3.35 ± 0.59	27.77 ± 3.79	10.40 ± 2.66
Hervéou et al., 2018 [35]	34.3 ± 5.9	3.2 ± 0.6	NR	−11.5 ± 4.0

*F_0_: maximal theoretical force, V_0_: maximal theoretical velocity, Pmax: maximal power output, HLT: High-Load Training fatigue protocol, LLB: Low-Load, High-Velocity fatigue protocol, FD/VD/WB: Force-Dominant/Velocity-Dominant/Well-Balanced training groups, NR: Not Reported.*

**Table 4:** Correlations, maturation effects, and key findings across studies.

Author (Year)	Correlations Reported	Key Findings
Zghal et al., 2025 [32]	NR	Vertical FV profile improved with maturation; increases in F0 and Pmax; differences disappear when normalized to body mass.
Bouvier et al., 2025 [37]	NR	Menstrual cycle and contraceptive use did not affect FV variables.
Li et al., 2024 [34]	NR	HLT reduced F0 more; LLB reduced V0 more; Pmax decreased similarly in both protocols.
Galantine et al., 2024 [33]	Only Mid-PHV significant: F0 ↔ SJ: r = 0.44; F0 ↔ CMJ: r = 0.48	F0 and Pmax increased with maturity; correlations only in less mature athletes.
Ben Hassen et al., 2024 [36]	PmaxVT ↔ Hmax: r = 0.58; V0VT ↔ Hmax: r = 0.35; Pmax ↔ V0: r = 0.85; others ns	WM and F show stronger power profiles; no positional differences in F0 due to variability.
Junge et al., 2021 [38]	Pmax ↔ Sprint FV: r = 0.73; Pmax ↔ Hip Thrust FV: r = 0.44; others ns	Pmax shows strongest cross-task associations; F0 and V0 show task-specificity.
Manson et al., 2021 [15]	V0 ↔ ACC: −0.32 to −0.44; CMJ ↔ MSS: r = −0.67; others small or NS	V0 and Pmax moderately linked to acceleration and MSS; F0 not related.
Fernández-Galván et al., 2021 [11]	F0 ↔ Maturity: r = 0.91; Pmax ↔ Maturity: r = 0.87; V0 ↔ Maturity: r = 0.23; Sfv ↔ Maturity: ns	F0 and Pmax show strong biological age associations; V0 weakly related; Sfv unrelated.
Marcote-Pequeño et al., 2018 [17]	Pmax ↔ SJ: r = 0.84; V0 ↔ SJ: significant; F0 ↔ SJ: ns; Sfv ↔ SJ: ns	Pmax strongly correlated with SJ height; FV profile is task-specific.
Hervéou et al., 2018 [35]	NR	Goalkeepers showed velocity-oriented FV profile with high V0 and lower F0.

*F_0_: Maximal theoretical force, V_0_: Maximal theoretical velocity, Pmax: maximal power output, Sfv: force–velocity slope, ACC: acceleration, MSS: Maximal Sprint Speed, CMJ: countermovement jump, SJ: squat jump, NR: Not Reported, VT/HZT: vertical task/horizontal task, ↔ indicates correlation between variable, r = Pearson correlation coefficient*.

**Table 5:** Summary of vertical force–velocity profile parameters across competitive levels.

Competitive Level	F_0_ (N·kg^−1^)	V_0_ (m·s^−1^)	Pmax (W·kg^−1^)	F–V Slope (N·s·m^−1^·kg^−1^)	Included Studies
Professional male players	36.0 ± 2.5	3.24 ± 0.53	31.0 ± 4.0	−11.5 ± 4.0	Hervéou et al., 2018 [35]; Junge 2021 et al., [38] (*n* = 2)
Elite youth male players (PHV/U10–U18)	24.5 ± 1.4	3.27 ± 0.86	20.2 ± 5.3	−7.9 ± 1.3	Galantine 2024 [33]; Fernández-Galván et al., 2021 [11]; Ben Hassen [36] (*n* = 3)
Sub-elite/Amateur males	28.3 ± 1.4	3.30 ± 0.84	22.8 ± 2.9	−9.3 ± 0.8	Zghal et al., 2025 [32] (*n* = 1)
Elite female players	33.2 ± 1.8	2.82 ± 0.40	23.7 ± 2.1	−12.6 ± 1.1	Marcote-Pequeño et al., 2018 [17]; Bouvier et al., 2025 [37]; Manson et al., 2021 [15] (*n* = 3)
Fatigue/intervention baseline values	48.2 ± 3.4	2.46 ± 0.11	29.7 ± 3.5	19.7 ± 1.5	Li et al., 2024 [34] (*n* = 1)

**Table 6:** Joanna Briggs Institute (JBI) risk-of-bias assessment across included studies (*n* = 10).

Study (Author, Year)	Q1	Q2	Q3	Q4	Q5	Q6	Q7	Q8	Overall Risk
Zghal et al., 2025 [32]	Yes	Yes	Yes	Yes	No	No	Yes	Yes	Moderate
Bouvier et al., 2025 [37]	Yes	Yes	Yes	Yes	Yes	Partial	Yes	Yes	Low
Li et al., 2024 [34]	Yes	Yes	Yes	Yes	Partial	No	Yes	Yes	Moderate
Galantine et al., 2024 [33]	Yes	Yes	Yes	Yes	Partial	Partial	Yes	Yes	Moderate
Ben Hassen et al., 2024 [36]	Yes	Yes	Yes	Yes	Partial	No	Yes	Yes	Moderate
Junge et al., 2021 [38]	Yes	Yes	Yes	Yes	Partial	No	Yes	Yes	Moderate
Manson et al., 2021 [15]	Yes	Yes	Yes	Yes	Partial	No	Yes	Yes	Moderate
Fernández-Galván et al., 2021 [11]	Yes	Yes	Yes	Yes	Yes	Yes	Yes	Yes	Low
Marcote-Pequeño et al., 2018 [17]	Yes	Yes	Yes	Yes	No	No	Yes	Yes	Moderate
Hervéou et al., 2018 [35]	Partial	Yes	Yes	Yes	No	No	Yes	Partial	Moderate

Q1: Were the criteria for inclusion in the sample clearly defined?/Q2: Were the study subjects and the setting described in detail?/Q3: Was the exposure measured in a valid and reliable way?/Q4: Were objective, standard criteria used for measurement of the condition?/Q5: Were confounding factors identified?/Q6: Were strategies to deal with confounding factors stated?/Q7: Were the outcomes measured in a valid and reliable way?/Q8: Was appropriate statistical analysis used?

## Data Availability

No new data were created or analyzed in this study. Data sharing is not applicable to this article.

## Supplementary Materials

The following supporting information can be downloaded at: https://www.mdpi.com/article/10.3390/jfmk11010099/s1, Supplementary File S1: Full Electronic Search Strategies.

## References

[1] Ribeiro J., Afonso J., Camões M., Sarmento H., Sá M., Lima R., Oliveira R., Clemente F.M. (2021). Methodological Characteristics, Physiological and Physical Effects, and Future Directions for Combined Training in Soccer: A Systematic Review. Healthcare.

[2] Stølen T., Chamari K., Castagna C., Wisløff U. (2005). Physiology of Soccer: An Update. Sports Med..

[3] Morin J.-B., Samozino P. (2016). Interpreting Power-Force-Velocity Profiles for Individualized and Specific Training. Int. J. Sports Physiol. Perform..

[4] Samozino P., Morin J.-B., Hintzy F., Belli A. (2008). A simple method for measuring force, velocity and power output during squat jump. J. Biomech..

[5] Lindberg K., Solberg P., Rønnestad B.R., Frank M.T., Larsen T., Abusdal G., Berntsen S., Paulsen G., Sveen O., Seynnes O. (2021). Should we individualize training based on force-velocity profiling to improve physical performance in athletes?. Scand. J. Med. Sci. Sports.

[6] Bobbert M.F., Lindberg K., Bjørnsen T., Solberg P., Paulsen G. (2023). The Force–Velocity Profile for Jumping: What It Is and What It Is Not. Med. Sci. Sports Exerc..

[7] Kotani Y., Lake J., Guppy S.N., Poon W., Nosaka K., Hori N., Haff G.G. (2022). Reliability of the Squat Jump Force-Velocity and Load-Velocity Profiles. J. Strength Cond. Res..

[8] Jonson A.M., Girard O., Wall B.A., Walden T.P., Goods P.S.R., Galna B., Scott B.R. (2024). Reliability and validity of free-weight countermovement jumps to characterize force-velocity-power profiles. Eur. J. Sport Sci..

[9] Ettema G. (2024). The force-velocity profiling concept for sprint running is a dead end. Int. J. Sports Physiol. Perform..

[10] Solberg P., Hopkins W.G., Andersen V., Lindberg K., Bjørnsen T., Saeterbakken A., Paulsen G. (2025). Force-velocity profile based training to improve vertical jump performance a systematic review and meta analysis. Sci. Rep..

[11] Fernandez-Galvan L.M., Boullosa D., Jimenez-Reyes P., Cuadrado-Penafiel V., Casado A. (2021). Examination of the sprinting and jumping force-velocity profiles in young soccer players at different maturational stages. Int. J. Environ. Res. Public Health.

[12] Kozinc Z., Zitnik J., Smajla D., Sarabon N. (2022). The difference between squat jump and countermovement jump in 770 male and female participants from different sports. Eur. J. Sport Sci..

[13] González-Jarrín P., Fernández-Fernández J., García-Tormo J.V., Gutiérrez García C. (2025). Neuromuscular Performance of High-Level Football Goalkeepers by Age Category and Sex: A Systematic Review. J. Funct. Morphol. Kinesiol..

[14] Lloyd R.S., Oliver J.L. (2012). The Youth Physical Development Model: A New Approach to Long-Term Athletic Development. Strength Cond. J..

[15] Manson S., Low C., Legg H., Patterson S., Meylan C. (2021). Vertical Force-velocity Profiling and Relationship to Sprinting in Elite Female Soccer Players. Int. J. Sports Med..

[16] Jiménez-Reyes P., Samozino P., Brughelli M., Morin J.-B. (2017). Effectiveness of an Individualized Training Based on Force-Velocity Profiling during Jumping. Front. Physiol..

[17] Marcote-Pequeño R., García-Ramos A., Cuadrado-Peñafiel V., González-Hernández J.M., Gómez M.Á., Jiménez-Reyes P. (2019). Association Between the Force–Velocity Profile and Performance Variables Obtained in Jumping and Sprinting in Elite Female Soccer Players. Int. J. Sports Physiol. Perform..

[18] Cross M.R., Brughelli M., Brown S.R., Samozino P., Gill N.D., Cronin J.B., Morin J.-B. (2015). Mechanical Properties of Sprinting in Elite Rugby Union and Rugby League. Int. J. Sports Physiol. Perform..

[19] García-Ramos A., Pérez-Castilla A., Jaric S. (2021). Optimisation of applied loads when using the two-point method for assessing the force-velocity relationship during vertical jumps. Sports Biomech..

[20] Šarabon N., Kozinc Ž., Marković G. (2020). Force–velocity profile during vertical jump cannot be assessed using only bodyweight jump and isometric maximal voluntary contraction tasks. Sci. Rep..

[21] Clemente F., Praça G.M., Aquino R., Castillo D., Raya-González J., Rico-González M., Afonso J., Sarmento H., Filipa Silva A., Silva R. (2023). Effects of pitch size on soccer players’ physiological, physical, technical, and tactical responses during small-sided games: A meta-analytical comparison. Biol. Sport.

[22] García-Pinillos F., Bujalance-Moreno P., Lago-Fuentes C., Ruiz-Alias S., Domínguez-Azpíroz I., Mecías-Calvo M., Ramirez-Campillo R. (2021). Effects of the Menstrual Cycle on Jumping, Sprinting and Force-Velocity Profiling in Resistance-Trained Women: A Preliminary Study. Int. J. Environ. Res. Public Health.

[23] Rumpf M.C., Lockie R.G., Cronin J.B., Jalilvand F. (2016). Effect of Different Sprint Training Methods on Sprint Performance Over Various Distances: A Brief Review. J. Strength Cond. Res..

[24] Ramirez-Campillo R., Thapa R.K., Afonso J., Perez-Castilla A., Bishop C., Byrne P.J., Granacher U. (2023). Effects of Plyometric Jump Training on the Reactive Strength Index in Healthy Individuals Across the Lifespan: A Systematic Review with Meta-analysis. Sports Med..

[25] Villamin P., Lopez V., Thapa D.K., Cleary M. (2025). A Worked Example of Qualitative Descriptive Design: A Step-by-Step Guide for Novice and Early Career Researchers. J. Adv. Nurs..

[26] Baena-Raya A., García-Mateo P., García-Ramos A., Rodríguez-Pérez M.A., Soriano-Maldonado A. (2022). Delineating the potential of the vertical and horizontal force-velocity profile for optimizing sport performance: A systematic review. J. Sports Sci..

[27] Gualtieri A., Rampinini E., Dello Iacono A., Beato M. (2023). High-speed running and sprinting in professional adult soccer: Current thresholds definition, match demands and training strategies. A systematic review. Front. Sports Act. Living.

[28] Page M.J., McKenzie J.E., Bossuyt P.M., Boutron I., Hoffmann T.C., Mulrow C.D., Shamseer L., Tetzlaff J.M., Moher D. (2021). Updating guidance for reporting systematic reviews: Development of the PRISMA 2020 statement. J. Clin. Epidemiol..

[29] Rethlefsen M.L., Page M.J. (2021). PRISMA 2020 and PRISMA-S: Common questions on tracking records and the flow diagram. J. Med. Libr. Assoc..

[30] Schiavo J.H. (2019). PROSPERO: An International Register of Systematic Review Protocols. Med. Ref. Serv. Q..

[31] Amir-Behghadami M., Janati A. (2020). Population, Intervention, Comparison, Outcomes and Study (PICOS) design as a framework to formulate eligibility criteria in systematic reviews. Emerg. Med. J..

[32] Zghal F., Rebai H., Colson S.S., Samozino P., Rahmani A., Peyrot N., Morin J.-B. (2025). Age-Related Differences in Jumping and Sprinting Performance and Force Production Capacities in Young Soccer Players. Eur. J. Sport Sci..

[33] Galantine P., Bertin D., Duché P., Hays A. (2024). Effect of maturity status on force-velocity relationships in a ballistic lower limb test in high-level soccer players. J. Sports Sci..

[34] Li Z., Zhi P., Yuan Z., García-Ramos A., King M. (2024). Feasibility of vertical force–velocity profiles to monitor changes in muscle function following different fatigue protocols. Eur. J. Appl. Physiol..

[35] Hervéou T., Rahmani A., Chorin F., Frère J., Ripamonti M., Durand S. (2018). Force-velocity muscular profiles and jumping performances of soccer goalkeeper. Sci. Sports.

[36] Ben Hassen D., Zghal F., Peyrot N., Samozino P., Rebaï H., Rahmani A. (2023). Jump and sprint force velocity profile of young soccer players differ according to playing position. J. Sports Sci..

[37] Bouvier J., Igonin P.-H., Boithias M., Fouré A., Belli A., Boisseau N., Martin C. (2025). The squat jump and sprint force–velocity profiles of elite female football players are not influenced by the menstrual cycle phases and oral contraceptive use. Eur. J. Appl. Physiol..

[38] Junge N., Lundsgaard A., Hansen M.F., Samozino P., Morin J.-B., Aagaard P., Contreras B., Nybo L. (2021). Force-velocity-power profiling of maximal effort sprinting, jumping and hip thrusting: Exploring the importance of force orientation specificity for assessing neuromuscular function. J. Sports Sci..

[39] Pareja-Blanco F., Sánchez-Medina L., Suárez-Arrones L., González-Badillo J.J. (2017). Effects of Velocity Loss During Resistance Training on Performance in Professional Soccer Players. Int. J. Sports Physiol. Perform..

[40] Samozino P., Edouard P., Sangnier S., Brughelli M., Gimenez P., Morin J.-B. (2013). Force-Velocity Profile: Imbalance Determination and Effect on Lower Limb Ballistic Performance. Int. J. Sports Med..

[41] Peña-González I., Javaloyes A., Cervelló E., Moya-Ramón M. (2022). The maturity status but not the relative age influences elite young football players’ physical performance. Sci. Med. Footb..

[42] Buchheit M., Mendez-Villanueva A. (2014). Effects of age, maturity and body dimensions on match running performance in highly trained under-15 soccer players. J. Sports Sci..

[43] Castillo D., Raya-González J., Manuel Clemente F., Yanci J. (2020). The influence of youth soccer players’ sprint performance on the different sided games’ external load using GPS devices. Res. Sports Med..

[44] Loturco I., Pereira L., Kobal R., Abad C., Komatsu W., Cunha R., Arliani G., Ejnisman B., Pochini A., Nakamura F. (2018). Functional Screening Tests: Interrelationships and Ability to Predict Vertical Jump Performance. Int. J. Sports Med..

[45] Jiménez-Reyes P., Samozino P., García-Ramos A., Cuadrado-Peñafiel V., Brughelli M., Morin J.-B. (2018). Relationship between vertical and horizontal force-velocity-power profiles in various sports and levels of practice. PeerJ.

[46] Morin J., Jiménez-Reyes P., Brughelli M., Samozino P. (2019). When Jump Height is not a Good Indicator of Lower Limb Maximal Power Output: Theoretical Demonstration, Experimental Evidence and Practical Solutions. Sports Med..

[47] Cotteret C., González-de-la-Flor Á., Prieto Bermejo J., Almazán Polo J., Jiménez Saiz S.L. (2025). A Narrative Review of the Velocity and Acceleration Profile in Football: The Influence of Playing Position. Sports.

[48] Marqués-Jiménez D., Calleja-González J., Arratibel-Imaz I., Terrados N. (2022). Match Loads May Predict Neuromuscular Fatigue and Intermittent-Running Endurance Capacity Decrement after a Soccer Match. Int. J. Environ. Res. Public Health.

[49] Sierra-Casas A., Rodríguez-Marroyo J.A., Castillo D., Gutiérrez-Arroyo J., Rodríguez-Fernández A. (2025). From load monitoring to training decisions: A practical approach using drop jump metrics in semi-professional soccer. BMC Sports Sci. Med. Rehabil..

[50] Jiménez-Reyes P., Samozino P., Morin J.-B. (2019). Optimized training for jumping performance using the force-velocity imbalance: Individual adaptation kinetics. PLoS ONE.

[51] Jiménez-Reyes P., Samozino P., Pareja-Blanco F., Conceição F., Cuadrado-Peñafiel V., González-Badillo J.J., Morin J.-B. (2017). Validity of a Simple Method for Measuring Force-Velocity-Power Profile in Countermovement Jump. Int. J. Sports Physiol. Perform..

[52] Ishihara R., Hill P., Short T., Cooke K., Sackmann T., Elms J., Yamada P. (2024). Utilizing force-velocity profiling to improve performance in collegiate American football players. Trends Sport Sci..

