# Supplementary material for: Vertical Force–Velocity Profiling in Soccer: A Systematic Review of Evidence, Assumptions, and Limitations
Source: J Funct Morphol Kinesiol. 2026 Feb 27;11(1):99. doi: 10.3390/jfmk11010099 (PMC13028409; doi:10.3390/jfmk11010099)
Supplement: Supplementary file 1 [file jfmk-11-00099-s001.zip › jfmk-4117291-supplementary.pdf]

Supplementary File S1

Full Electronic Search Strategies

General Parameters

- Databases searched: Scopus, Web of Science Core Collection, PubMed, ScienceDirect
- Search period: January 2015 to April 2025
- Last search date: 15 April 2025
- Language restriction: English
- Document type: Peer-reviewed journal articles
- Population: Male and female soccer (association football) players

1. Scopus

Search string:

(TITLE-ABS-KEY("football" OR "soccer"))

AND

(TITLE-ABS-KEY("force-velocity profile"

OR "vertical force-velocity profiling"

OR "vertical force-velocity profile"

OR "F-V profile"

OR "force velocity profiling"

OR "FVP"))

Filters applied:

- Publication years: 2015–2025
- Language: English
- Document type: Article

2. Web of Science Core Collection

Search string:

TS=("football" OR "soccer")

AND

TS=("force-velocity profile"

OR "vertical force-velocity profiling"

OR "vertical force-velocity profile"

OR "F-V profile"

OR "force velocity profiling"

OR "FVP")

Filters applied:

- Timespan: 2015–2025
- Language: English
- Document types: Article

3. PubMed

Search string:

(("football"[Title/Abstract] OR "soccer"[Title/Abstract])

AND

("force-velocity profile"[Title/Abstract]

OR "vertical force-velocity profiling"[Title/Abstract]

OR "vertical force-velocity profile"[Title/Abstract]

OR "F-V profile"[Title/Abstract]

OR "force velocity profiling"[Title/Abstract]

OR "FVP"[Title/Abstract]))

Filters applied:

- Publication dates: 2015/01/01 to 2025/04/15
- Language: English
- Article type: Journal Article

4. ScienceDirect

Search performed using the advanced search interface with the following terms:

("football" OR "soccer")

AND

("force-velocity profile"

OR "vertical force-velocity profiling"

OR "vertical force-velocity profile"

OR "F-V profile"

OR "force velocity profiling"

OR "FVP")

Filters applied:

- Research articles

- Publication years: 2015–2025
- English language

Additional Search Procedures

- Manual screening of reference lists of included articles
- No additional eligible studies were identified beyond database searches
- Search strategies reported in accordance with PRISMA-S guidelines
